# Biofilm associated with pigmented areas on a waterproofing coating surface

**DOI:** 10.3934/microbiol.2025005

**Published:** 2025-01-25

**Authors:** Clotilde Maestri, Ronan L. Hébert, Patrick Di Martino

**Affiliations:** 1 Laboratoire ERRMECe, Cergy Paris University, 1 Rue Descartes, 95000 Neuville-sur-Oise, France; 2 Laboratoire GEC, Cergy Paris University, 1 Rue Descartes, 95000 Neuville-sur-Oise, France

**Keywords:** biodegradation, discolouration, coating, varnish, polyurethane, biofilm

## Abstract

Waterproofing coatings are composite materials made of different layers with complementary functionalities. They may suffer damage that can modify their aesthetic appearance and/or their functionality. In this study, dark stains appearing on a waterproofing coating of a public swimming pool were mapped and characterized at a macroscopic scale through visual observation and by colorimetric analysis, as well as at a microscopic scale with a digital microscope, a confocal laser scanning microscope, and a scanning electron microscope. Five stains were differentiated macroscopically and characterized using colorimetry and principal component analysis. Microscopic observations showed the presence of microorganisms of varied morphology, some filamentous but mostly unicellular. Biofilms consisting of ovoid fluorescent cells with the morphology of Chlorophyta and unicellular cyanobacteria were particularly abundant. The pigmented stains were located at top coat disorders where microbial colonization and biofilm development were observed. The microscopic observations suggested that physical degradation of the surface of the material would have constituted a prerequisite for colonization by pigmented microorganisms which would have led to the development of macroscopically visible pigmented areas. In this case study, the damage remained superficial and did not alter the watertightness of the material so far.

## 1. Introduction

Concrete is a permeable and porous material that must be protected from humidity especially in contexts such as roof terrace or pools [1]. Water infiltration can reduce the lifespan of concrete through deterioration, in particular due to concrete permeability increase resulting from porosity enhancement and crack formation that facilitate water leaks and cause corrosion of the metal reinforcements of the structure. Waterproofing is a common approach to preventing water damage in cement construction [2]. Some waterproofing coatings are composite materials made up of different layers with complementary functionalities. The deepest layers ensure the mechanical and waterproofing properties of the composite material. The outermost layer is a finishing layer directly exposed to humidity, chemical, mechanical, and physical aggressions. For outdoor construction, this finishing layer is subject to weathering and undergoes wind, rain, temperature changes, and UV irradiation. It also suffers from the use of chemicals for disinfection, cleaning, abrasion, and impacts caused by mechanical cleaning, or by carts and pedestrian traffic. Polyurethanes (PUs) are polymers widely used for waterproofing applications [3]. These PUs are not affected by water or many chemicals; they are elastic and not very sensitive to abrasion phenomena and to biodegradation. However, many microorganisms and moulds can biodegrade PUs by enzymatic hydrolysis [4].

The appearance of dark stain on buildings can have several causes [5]. If the appearance of these pigmented areas can be linked to physicochemical phenomena and atmospheric pollution (e.g., black crusting due to road traffic fine particles), it is often of biological origin [6]–[8]. Discoloured surfaces constitute aesthetic damage requiring cleaning when possible. Furthermore, if the appearance of pigmentation is linked to microbial colonization and biofilm development, it can be associated with physicochemical alterations by biodegradation processes [9],[10].

This work was a case study of a swimming pool waterproofing coating with a polyurethane topcoat that exhibits various blackish spots. The dark stains were analysed using macroscopic and microscopic approaches to determine their origin. Microbial colonization and material degradation associated with the blackish spots were sought after.

## 2. Materials and methods

### 2.1. Sampling

**Figure 1.**
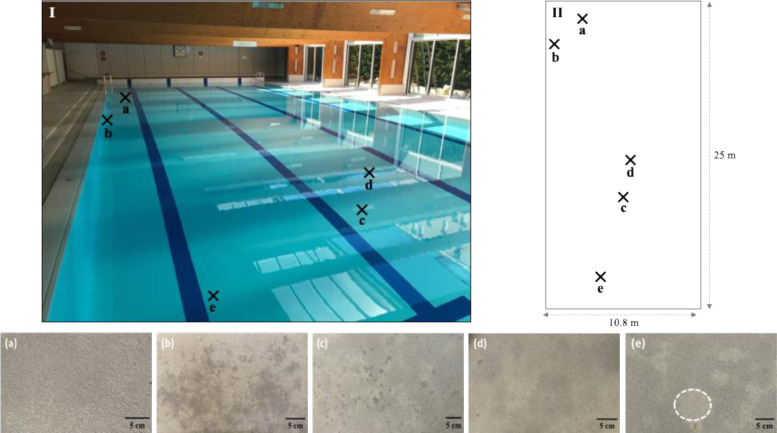
Location of stained areas taken from the basin. I, image of the swimming pool basin. II, schematic representation of the swimming pool basin. a, blackish stain extending on the slab. b, blackish stain extending at the slab/wall junction. c, isolated black spots scattered on the slab. d, black spots extended on the slab. e, healthy area without apparent stain. The precise healthy area sampled is surrounded by white dotted lines.

The study site was the basin of a Caneton swimming pool. The Caneton swimming pool model came from a national program to build public swimming pools in France from the late 1970s to the early 1980s. The waterproof coating of the swimming pool was a composite material with three major layers. From bottom to top: (i) The reinforced base layer, which ensures waterproofing that is in contact with the concrete of the pool structure; (ii) the base layer made of quartz grains is incorporated into an epoxy resin; and (iii) the finishing layer, which is composed of polyurethane varnish that covers the whole, for a total thickness of 4 ± 0,5 mm. The pigmented spots were mapped and classified according to several visual criteria: Location on the slab and/or the wall, colour, density, and size. Four stain types and one “healthy area” (i.e., with no evidence of colour change to be used as reference sample) were collected (Figure 1).

The samples were taken using a chainsaw depending on their location and extent. The sampled area was removed from the support using a hammer and chisel, and then stored in a plastic bag. All samples measured either 5 × 5 cm or 5 × 15 cm and had an average thickness of 5 mm. The samples brought to the laboratory were stored away from light at + 4 °C for less than 3 days before processing and analysis. In the laboratory, the samples were fixed in a bath of 4% paraformaldehyde (PFA) (Sigma-Aldrich) for 1 hour followed by three successive rinses with distilled water. After fixation, the samples were cut into pieces of 1 to 2 cm sides using a circular saw to facilitate subsequent analysis.

### 2.2. Microscopic analysis

#### 2.2.1. Optical microscopy

Observations of the surface of the coating and the microbial colonization of the samples were first carried out at the microscopic scale with a digital microscope (Olympus/DSX110). The PFA-fixed samples were directly observed.

#### 2.2.2. Scanning electron microscopy

The PFA-fixed samples were dehydrated by successive baths in ethanol at increasing percentages (30%, 50%, 80%, and 100%) for 10 minutes each, then dried at 27 °C overnight to evaporate the residues of ethanol. The samples were then metallized with platinum (Leica ACE600 metallizer) with an average thickness of 4 nm to make them conductive. The observations were carried out with a Scanning Electron Microscope (SEM, Zeiss/GeminiSEM 300) under partial vacuum with a voltage between 1 and 5 kV, depending on the samples.

#### 2.2.3. Confocal laser scanning microscopy

The PFA-fixed samples were directly observed under an inverted laser scanning confocal microscope (Zeiss/LSM900) for visualization of auto-fluorescent microorganisms. The excitation lengths used were 405 and 561 nm in order to reveal the fluorescence emission from chitin and cellulose or chlorophylls, respectively [11]–[13].

#### 2.2.4. Colorimetry

The surface colour of the samples was measured according to the L*a*b* system using a portable spectrophotometer (Konica Minolta/CM 2300d). A total of 10 measurements at different points were taken for each sample, with a measuring diameter of 8 mm. The colour E of the samples was calculated according to the formula: $E=\;\sqrt{{\left(L^{*}\right)}^{2}+\;{\left(a^{*}\right)}^{2}+\;{\left(b^{*}\right)}^{2}}$. The colour difference between each sample (2) and the healthy area showing no visible colour change (1) was calculated according to the formula: $E_{ab}^{*}=\;\sqrt{{\left(\Delta L^{*}\right)}^{2}+\;{\left(\Delta a^{*}\right)}^{2}+\;{\left(\Delta b^{*}\right)}^{2}}$ where $\Delta L^{*}=L_{2}^{*}\;-L_{1}^{*}$, $\Delta a^{*}=a_{2}^{*}\;-a_{1}^{*}$, $\Delta b^{*}=b_{2}^{*}\;-b_{1}^{*}$. The colorimetry data were analysed by Principal Component Analysis and student test.

## 3. Results

### 3.1. Colour analysis of samples

Visual observation of the pool basin revealed the presence of more or less extensive dark stains over the slab, at the junction between the slab and the walls, locally on the walls, and locally on the edges of the basin. To take into account the heterogeneity of the dark spots in terms of location and extent, five representative areas were sampled (Figure 1).

Based on the colorimetric analysis of the samples, the colour value E and the ΔE*ab value, representing the colour difference between each blackish sample (a, b, c or d) and the healthy sample (e), were calculated (Table 1).

**Table 1.** Data of the colorimetric analyses of the samples.

Samples	E +/- SD	E*ab
a	68.6 +/- 1.6***‡‡##††	8.6
b	73.3 +/- 3.5*###	3.93
c	65.7 +/- 1.7***‡‡‡††††	10.58
d	71.3 +/- 1.2***###	8.54
e	76.1 +/- 0.8‡###††††	0

a, blackish area extended on the slab. b, blackish area extended at the slab/wall junction. c, isolated black spots scattered on the slab. d, black spots extended on the slab. e, healthy area without apparent stain. SD: standard deviation. ***, *: The value differs significantly (*P* < 0.0001 and *P* < 0.05, respectively) from the value of the healthy area. ‡‡‡, ‡‡, ‡: The value differs significantly (*P* < 0.0001, *P* < 0.01, *P* < 0.05, respectively) from the value of sample b; ###, ##: The value differs significantly (*P* < 0.0001 and *P* < 0.01, respectively) from the value of sample c; ††††, ††: The value differs significantly (*P* < 0.01 and *P* < 0.05, respectively) from the value of sample d.

The E value of each of the four dark samples was statistically different from the value of the reference sample. The values of E for samples b and d were not statistically different from each other while the values of the other samples were significantly different from each other in pairs.

**Figure 2.**
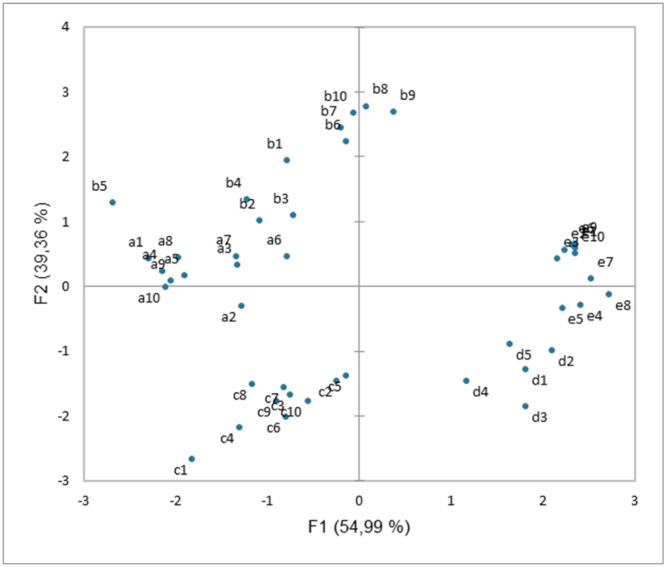
Principal Component Analysis (PCA) on the raw data from the colorimetric measurements (L*, a* and b*) of the five samples. a, blackish area extended on the slab. b, blackish area extended at the slab/wall junction. c, isolated black spots scattered on the slab. d, black spots extended on the slab. e, healthy area without apparent stain.

A Principal Component Analysis (PCA) carried out on the raw data of the colorimetric measurements (L*, a* and b*) is shown in Figure 2. The graphical representation of the PCA showed that the five samples presented different colorimetric data, the data from each sample was grouped together distinctly from the data from the other samples. The data from samples a and b were close, as were the data from samples d and e, while the data from sample c were isolated.

### 3.2. Analysis of dark stains at the microscopic scale

**Figure 3.**
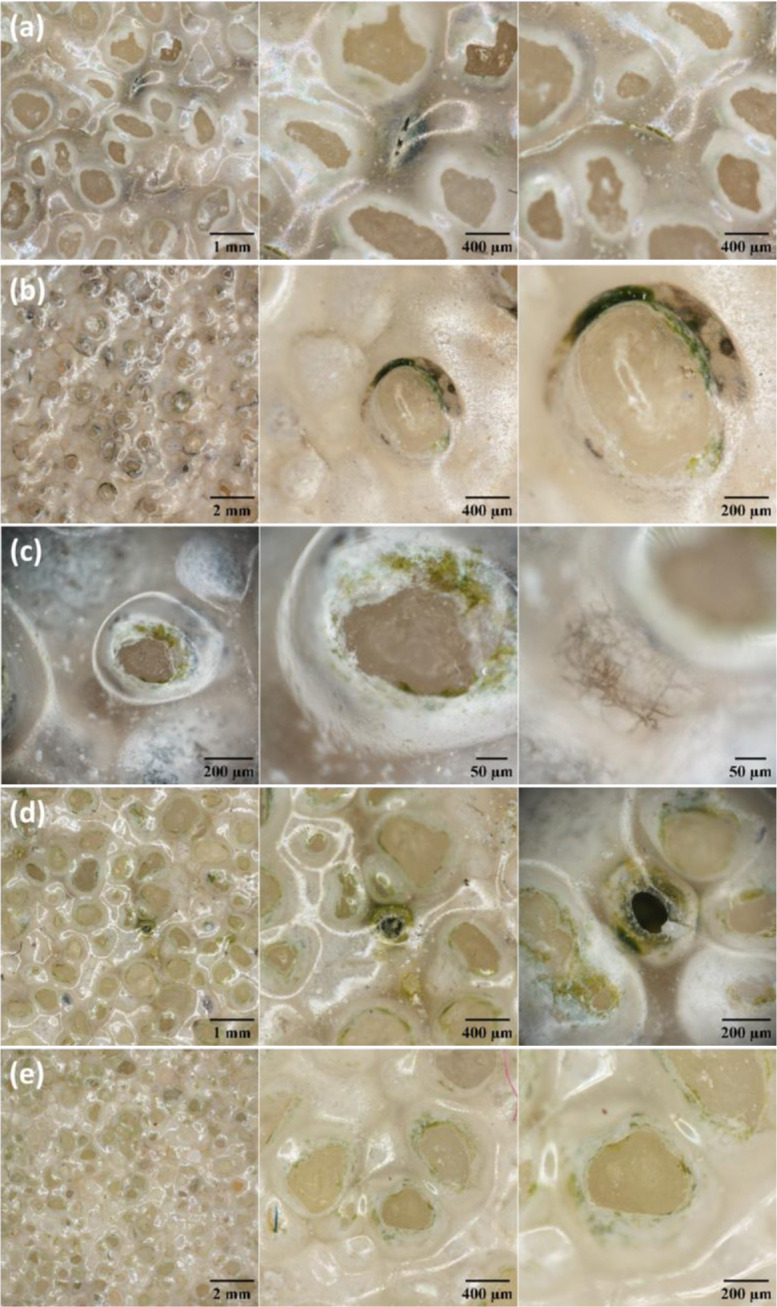
Observations of the different samples with a digital microscope. a, blackish area extended on the slab. b, blackish area extended at the slab/wall junction. c, isolated black spots scattered on the slab. d, black spots extended on the slab. e, healthy area without apparent stain.

The microscopic study was first carried out with a digital microscope to observe the surface of the samples, as well as the interior of the finishing layer through transparency (Figure 3).

A widespread superficial alteration of the finishing varnish was observed for all samples with the head of the quartz grains, which was no longer covered with varnish and the presence of green and/or black pigmentation at the grain-varnish interface (Figure 3a, b, c, d, e). Reference sample e, which was considered “healthy” at the macroscale, showed also disorder evidences at the microscopic scale (Figure 3e). Tangled black filaments were also observed in places under the varnish, near quartz grains, but not at the grain-varnish interface (Figure 3c). Other disorders of the varnish were observed away from the quartz grains, such as cracks or holes (Figure 3a and d, respectively). Here, again, green and black pigmentations were observed around and under the cracks and holes of the varnish.

**Figure 4.**
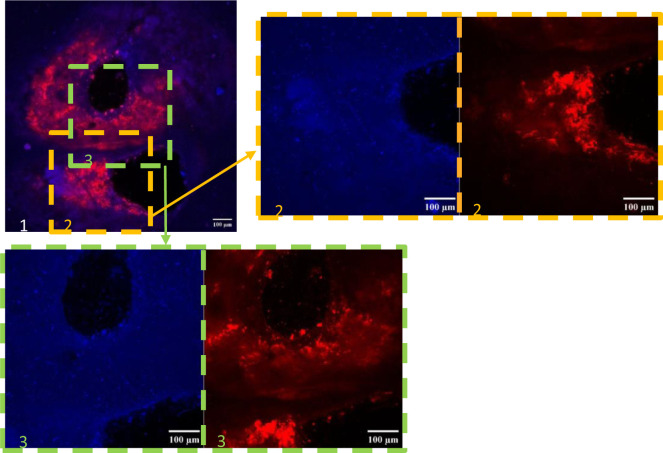
Observation of a pigmented zone on the surface of sample b (blackish zone extended to the slab/wall junction) using a confocal laser scanning microscope. 1, Superposition of images obtained after excitation at 405 nm (blue) and 561 nm (red). Figures 2 and 3, Zoom of areas of image 1 with separation of the images obtained after excitation at each of the wavelengths used (2a and 3a, excitation at 405 nm; 2b and 3b, excitation at 561 nm).

The presence and location of autofluorescent microorganisms at the level of the varnish were then sought by confocal laser scanning microscopy without specific labelling (Figure 4).

The blue fluorescence observed after excitation at 405 nm was weakly present throughout the resin but was concentrated in scattered precise points mainly around the quartz grains (Figure 4.2a and 4.3a). There were none on top of the quartz grains. The red fluorescence observed after excitation at 561 nm was observed only on the edge of the grains. It was intense, heterogeneous, and extended approximately 200 to 300 µm around the grains (Figure 4.2b and 4.3b). The surface of the quartz grains, which was no longer covered by varnish, showed no fluorescence.

**Figure 5.**
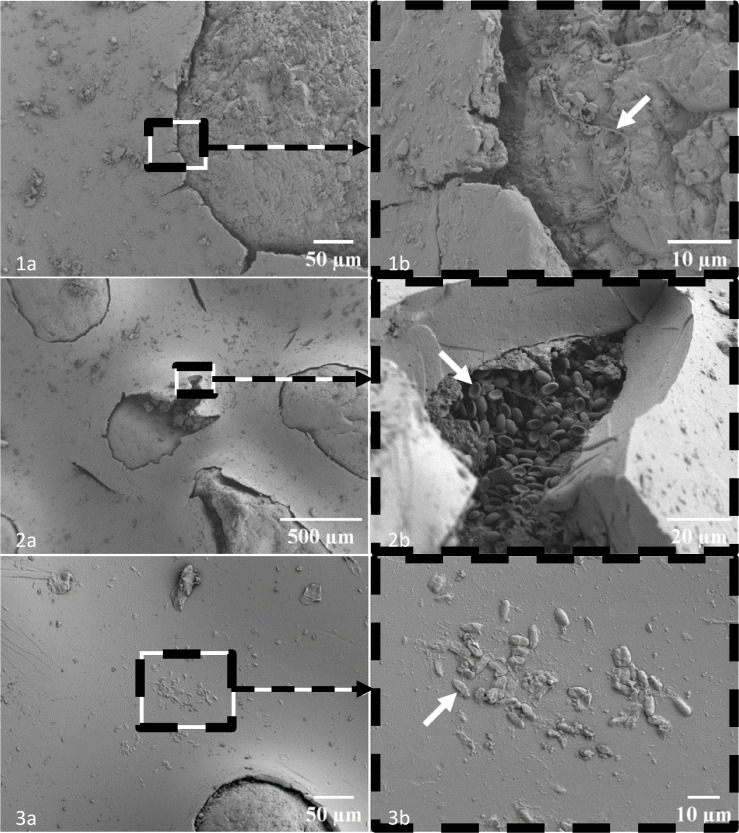
Observation of the surface alterations of the finishing varnish by scanning electron microscopy. 1, 2, 3: Altered areas of different samples. a, global views of the varnish-quartz grain interface. b, zoom of areas of photos a showing microbial colonization.

The surface alterations of the finishing varnish and the morphology of the microorganisms on its surface and at the varnish-quartz grain interface were also observed by scanning electron microscopy (Figure 5).

Numerous and varied superficial alterations of the finishing varnish were observed for all samples (Figure 5.1a, 5.2a, 5.3a). We observed that the interface between the varnish and the quartz grains was discontinuous with the appearance of multiple radial cracks (Figure 5.1a and 5.1b). Isolated cracks also appeared without direct relation to the grains (Figure 5.2a). Different microorganisms colonizing the surface of the varnish and in particular the varnish-quartz grain interfaces were observed. Filaments were present, sometimes on the surface of the quartz grains (Figure 5.1b). Ovoid cells with a size of approximately 5 µm formed a dense biofilm at the varnish-quartz grain interface (Figure 5.2b). A number of these cells presented a concavity linked to deformation following dehydration during sample preparation. Bacterial rod cells were observed in the form of microcolonies in unaltered areas of the varnish (Figure 5.3a and 5.3b).

## 4. Discussion

We report on a case study of visual disorders on a waterproof covering of a public swimming pool. The macroscopically disorders consisted of dark stains more or less widespread in different areas of the basin. The colorimetric analysis of samples taken made it possible to confirm the discrimination of the four categories of coloured areas characterized visually. The PCA of the colorimetry data made it possible to group together some of these categories, which visual observation had not enabled.

At the macroscopic level, the pigmented stains appeared dark and rather black, while at the microscopic level, the pigmentation observed was predominantly green and partially black. This difference between the two observation scales may be linked to the origin of the pigmentation. The green colour comes from the chlorophyll present in phototrophic microorganisms (Chlorophyta microalgae and cyanobacteria). Observations of autofluorescence made by confocal laser scanning microscopy and the morphology of cells within biofilms at the quartz grains-resin interface made by SEM show colonization by unicellular ovoid organisms of about 5 micrometres in length. This cellular morphology may correspond to Chlorophyta microalgae or to unicellular cyanobacteria previously called blue-green algae such as *Gloeocapsa* sp. [14]–[17]. Many cyanobacteria produce an extracellular yellow-brown fluorescent indole alkaloid pigment, scytonemine, that is involved in the blackening of building walls and roofs [18]–[20]. These cyanobacteria are particularly predominant in tropical environments with successive periods of strong sunshine, drought, and heavy rain, while microalgae are predominant in temperate climates such as in France [21]. In addition to sunlight, artificial lighting in an indoor pool, like the one in the study, should also promote the growth of phototrophic microorganisms [22]. This light, more uniform than natural light, should stimulate growth on all the walls of the basin. The involvement of microalgae in the greening of monuments is well documented [23],[24]. The formation of algal biofilm creates areas highly favourable for trapping particles that darken the surface of the material over time [25],[26]. These phenomena, well described for the facade and roofs of buildings, also seem to be involved in the blackening of polymer materials such as varnishes used as a finishing layer for waterproofing coatings.

Phototrophic microorganisms are not only responsible for the discoloration of materials exposed outdoor, but they play an active role in their physicochemical biodegradation [27],[28]. Mixed algae cultures of *Coenochloris signiensis*, *Stichococcus bacillaris*, *Klebsormidium flaccidum*, and *Chlorococcum infusionum* in biofilm carried out in the laboratory showed their potential for biodegradation of bricks and plaster. The development of mixed microalgae biofilms induced a change in the colour of the bricks, a decrease in the pH of the plaster, a production of acid metabolites, and an increase in the water absorption capacity of both materials [28]. Moreover, Chlorophyta and cyanobacteria biofilms exhibit large volume changes during drying and humidification cycles, which exerts powerful mechanical stress on the colonized material [29].

Microscopic observations of the varnish surface showed that colour change was not the only type of material disorder. Cracks and voids at the interface between the varnish and the quartz grains were observed. The formation of shrinkage cracks or structural cracks is a common problem in swimming pool materials [30]. In addition, the surface of many quartz grains was no longer covered with varnish. This damage does not seem to have a microbial origin and could be the result of mechanical shock and abrasion or chemical degradation linked to chlorine [31]. Although they are effective in protecting a concrete surface in contact with chlorinated water, polyurethane coatings are known for their limited resistance to degradation by mechanical stress [32]–[34]. Furthermore, polyether urethanes have been shown to be susceptible to chlorine attack through a degradation mechanism involving chain cleavages [35]. Chemical degradation by chlorine could weaken the polyurethane varnish, which would be more sensitive to physical degradation phenomena, particularly at the level of the top of the quartz grains.

## 5. Conclusions

To conclude, the microbial colonization of the waterproofing layer of the swimming pool studied was observed mainly in the degraded areas of the varnish. Since these degradations are most certainly not of biological origin, they would increase the bioreceptivity of the varnish by creating areas suitable for the development of biofilm by different microorganisms but mainly by unicellular algae and cyanobacteria, which would cause discoloration of the material.

## Use of AI tools declaration

The authors declare they have not used Artificial Intelligence (AI) tools in the creation of this article.
